# Fine-Needle Aspiration of an Incidental Pheochromocytoma Mimicking Renal Carcinoma: A Case Report and Review of Safety Considerations

**DOI:** 10.7759/cureus.99100

**Published:** 2025-12-13

**Authors:** Deepshikha Verma, Tina Rai, Pragati Awasthi, Abhiram Awasthi

**Affiliations:** 1 Pathology, Atal Bihari Vajpayee Government Medical College, Vidisha, IND; 2 Orthopedics and Traumatology, Jawaharlal Nehru Medical College, Wardha, IND

**Keywords:** cytology, fna cytology, guided fna, pheochromocytoma, renal carcinoma

## Abstract

Fine needle aspiration (FNA) of abdominal and intra-abdominal masses has been a safer and well-established procedure associated with minimal risk and low mortality. However, there have been limited reports in the literature regarding aspiration of adrenal masses. Radiologically guided fine-needle aspiration cytology (FNAC) has been performed in patients with malignant diseases or those with suspected adrenal malignancy and metastases. As FNA carries a risk of triggering a hypertensive crisis, it has generally been considered contraindicated in clinically suspected pheochromocytomas. However, the literature indicates that percutaneous tissue sampling of adrenal lesions is infrequent and is performed only selectively; adrenal incidentalomas are detected in ~1-5% of abdominal computed tomography (CT) examinations, while pheochromocytoma incidence is low (≈2-8 cases per million per year) and is present in an estimated 0.1-0.6% of hypertensive patients; therefore, routine FNA of adrenal masses is uncommon. Here, we present a known hypertensive case of an 82-year-old male who presented to the surgery department of our hospital with complaints of headache, urinary discomfort, and dizziness. Radiological findings suggested a suprarenal mass on the superior pole of the kidney that appeared neoplastic in origin. Guided FNAC was advised as clinicians primarily suspected renal carcinoma. FNA smears revealed tumor cells arranged in loosely cohesive clusters and scattered singly. These tumor cells were pleomorphic and showed prominent anisokaryosis, abundant eosinophilic cytoplasm, and intranuclear inclusions. Based on radiological and cytological findings, a diagnosis of pheochromocytoma was suggested and later confirmed histologically. FNA cytology is a low-risk tool that can provide diagnostic and prognostic information. However, due to the potentially fatal risk of hemorrhage and catecholamine release, its application in diagnosing adrenal tumors remains contentious. FNA in cases of pheochromocytoma is not always contraindicated, but aspiration must be conducted with utmost caution in a setting equipped to manage a hypertensive crisis.

## Introduction

Pheochromocytoma is a rare tumour of the adrenal medulla that produces catecholamines. It is usually found in adults, typically in the fourth to sixth decades of life, although it can occur at any age with a slight female predilection. Its clinical hallmark is sustained or intermittent hypertension, although it is a rare cause of hypertension [1]. Previously known as the "10% tumour," i.e., 10% extra-adrenal (commonly in the organ of Zuckerkandl), 10% bilateral, 10% malignant, 10% asymptomatic (normotensive), and 10% syndromic, it is now known that more than 25% are syndromic. The diagnosis is critical due to its potentially life-threatening consequences.

Adrenal pheochromocytomas are rare and account for only 1.5-11% of incidentally discovered adrenal tumors, although they remain the most common adrenal medullary tumors. Most patients present with symptoms such as headache, palpitations, anxiety, vomiting, weakness, or syncope [2].

In this paper, we focus on a case of pheochromocytoma diagnosed incidentally in an 82-year-old male who presented with complaints of headache, dizziness, and urinary discomfort.

Although FNA cytology of abdominal and intra-abdominal masses has been widely recognized as a safe and effective diagnostic tool, there are limited reports discussing its role in adrenal masses. It has generally been avoided in cases of suspected pheochromocytoma due to the risk of precipitating a hypertensive crisis. However, the literature suggests that this complication is rare.

## Case presentation

An 82-year-old male, with a history of hypertension for the past six months, presented to the surgery department with complaints of a burning sensation in the stomach, increased urinary frequency over the past month, and headache and dizziness for the past five days. A provisional diagnosis of a left renal cyst had previously been made, but renal function tests remained within normal limits.

**Figure 1:**
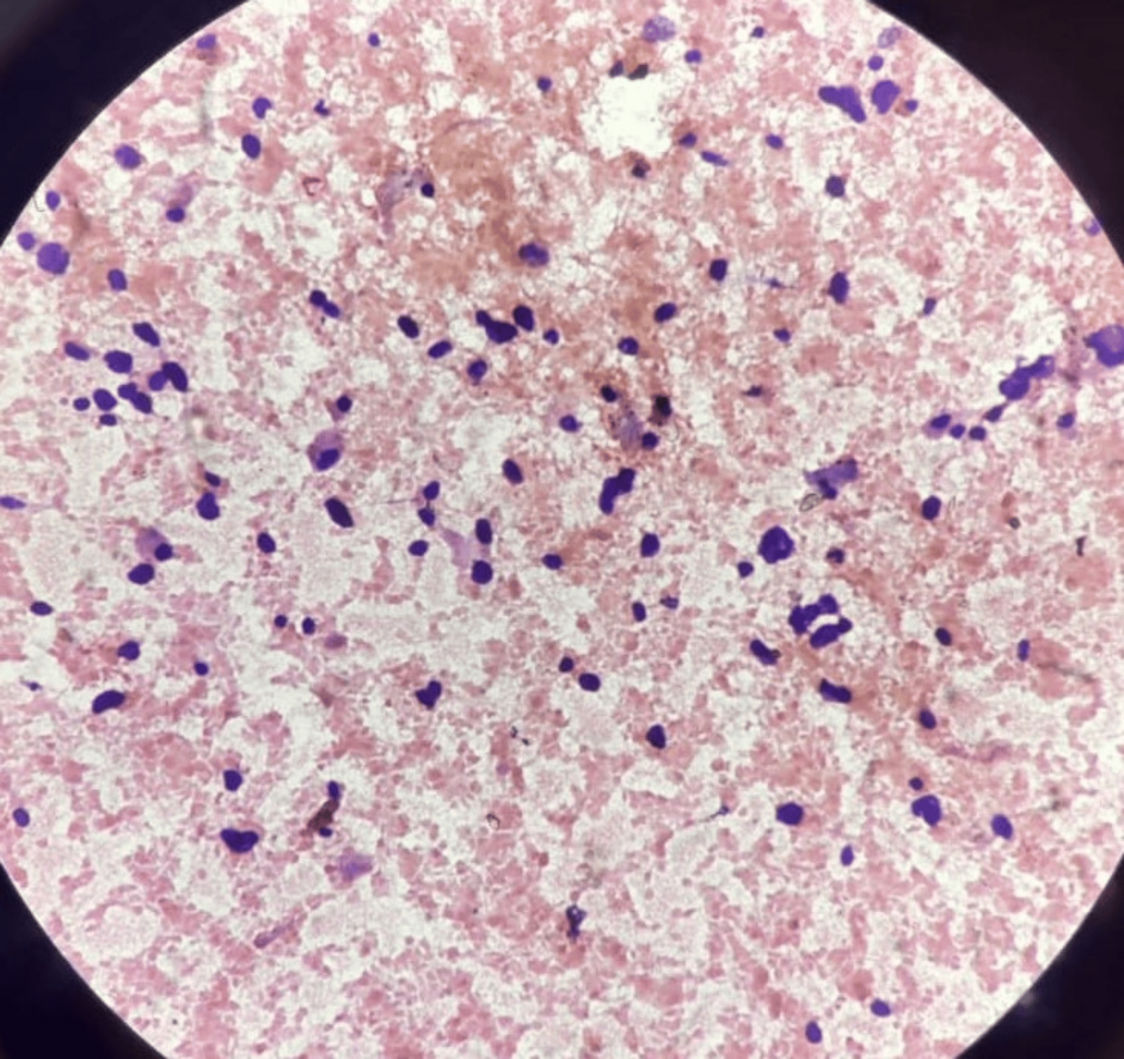
Low-power image (10x): Cytological smear shows predominantly dispersed tumour cells with coarse granular nuclear chromatin and prominent nucleoli along with abundant eosinophilic granular cytoplasm

**Figure 2:**
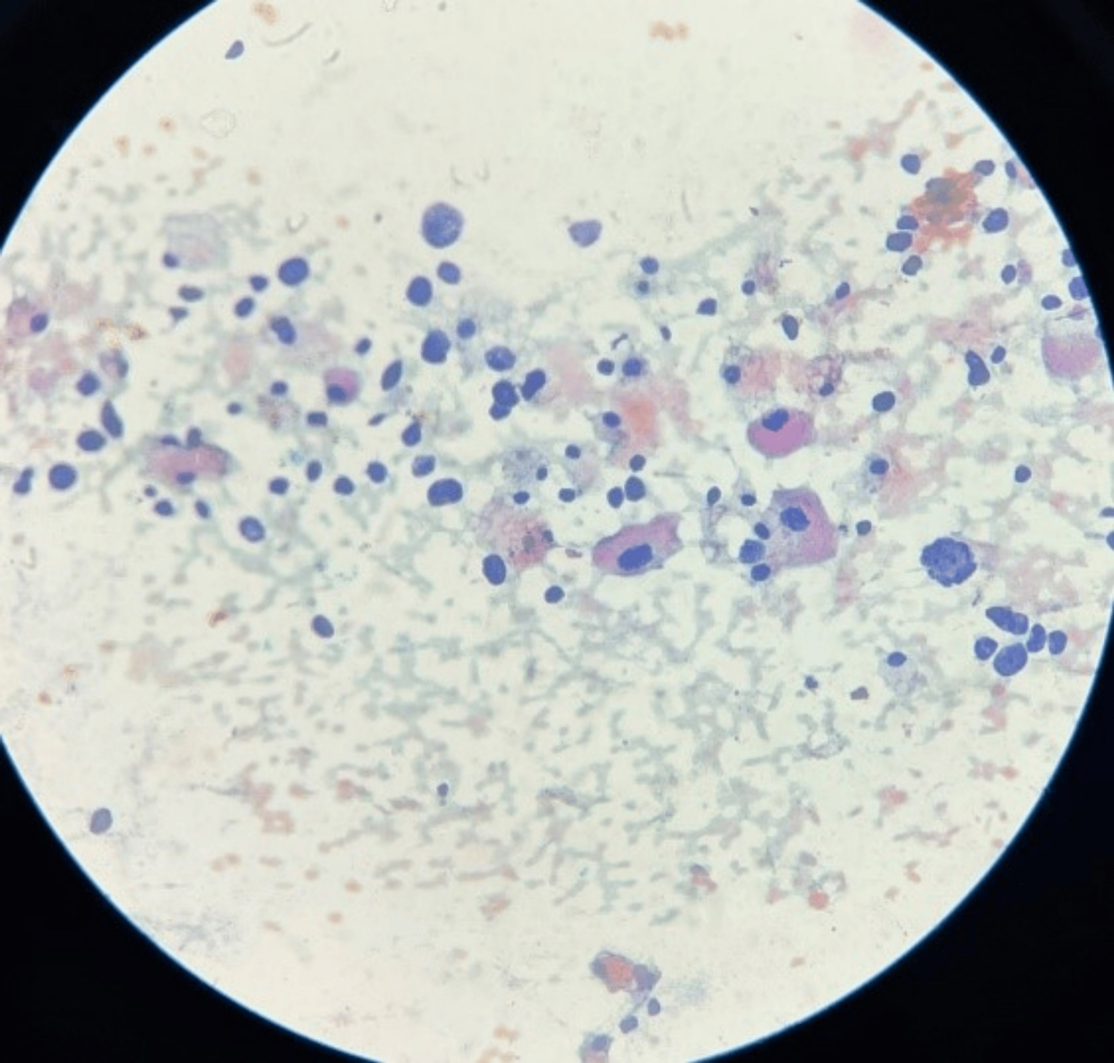
Low-power image (10x): Cytological smear shows cytoplasm of the cell appears to be fragile and cytoplasmic granules are seen dispersed in the background of haemorrhage. A few cells are also showing intranuclear inclusions.

Radiological examination revealed a mass in the suprarenal region at the upper pole of the kidney, suspected to be neoplastic in origin. Based on a preliminary diagnosis of renal carcinoma, an image-guided FNAC was performed. Cytological smears were found to be moderately cellular, with predominantly dispersed, scattered neoplastic cells and loosely clustered cells. In some areas, the tumor cells were arranged in small groups and micro-acinar patterns. The cells were round to oval in shape, with eccentrically placed nuclei and moderate to abundant eosinophilic granular cytoplasm. Marked pleomorphism and anisonucleosis were noted, along with coarse granular chromatin, prominent nucleoli, and intranuclear inclusions. Some cytoplasmic granules appeared to be fragile and were seen scattered in a hemorrhagic background in Pap stain (Figures 1, 2).

These features were cytomorphologically suggestive of pheochromocytoma. Histopathological correlation was advised for confirmation.

Differential diagnoses included oncocytoma, renal cell carcinoma (clear cell and chromophobe variants), adrenal cortical carcinoma, and neuroblastoma. These were ruled out based on cellular morphology, and a final diagnosis of pheochromocytoma was made.

Histological analysis revealed a well-encapsulated tumor composed of round to polygonal cells with eosinophilic granular cytoplasm and round vesicular nuclei. The tumor exhibited a classic Zellballen pattern-cells arranged in cords and nests surrounded by sustentacular cells and separated by blood capillaries and delicate fibrous septa. Some areas showed ribbons or trabecular arrangements, and hemorrhagic foci were also noted. Based on these features, a diagnosis of pheochromocytoma arising from the right adrenal gland was confirmed.

## Discussion

Pheochromocytoma was first recognized in 1926 by Casor Roux and Charles Mayo [2,3]. Pheochromocytoma and the related tumors, paragangliomas, belong to the group of tumors derived from the neural crest. Adrenal pheochromocytoma is the most common member of this family of tumors. Previously, it was called a chromaffinoma because of the brown staining of these tumors with salts of chromium. The annual incidence is 1-4/10^6^ population/year [4]. These tumors are important because they give rise to sudden attacks of hypertension [5]. Although most of the tumors of this family are benign in nature, about 10% of pheochromocytomas are malignant, and local invasion into surrounding tissues and organs or distant metastases is the only reliable clue for malignancy [6]. FNA cytology is a low-risk tool that can be commonly used to get diagnostic information. On the other hand, it may provide prognostic information too. However, because of the potentially fatal risk of hemorrhaging within the lesion, its application in diagnosing adrenal tumors is contentious. Moreover, there is not enough literature on research that addresses the cytologic characteristics of pheochromocytoma.

Furthermore, when pheochromocytoma is clinically unsuspected due to the lack of a typical presentation, FNA is often the modality used for the initial work-up [7].

Percutaneous sampling of adrenal lesions carries specific risks when catecholamine-secreting tumors are present. Reported complications include hypertensive crisis, severe hemorrhage, arrhythmia, and rare fatalities; most evidence derives from case reports and small series rather than large prospective cohorts. [8] Given the potentially catastrophic cardiovascular sequelae of catecholamine surge, current clinical practice guidelines and multiple reviews recommend biochemical exclusion of pheochromocytoma before attempting invasive tissue sampling of an adrenal lesion unless there is a compelling oncologic indication and the result would change management [9].

When FNA or core biopsy is considered unavoidable (for example, suspected metastatic disease in a patient with known extra-adrenal primary), the procedure should be planned in a controlled setting: pre-procedural biochemical screening (plasma free metanephrines or urinary fractionated metanephrines) when feasible; peri-procedural monitoring; availability of intravenous antihypertensive agents and experienced anesthesia/critical care support; and the lowest necessary needle passes and gauge. Case reports of biopsy-related catecholamine crises following endoscopic ultrasound (EUS)-guided sampling and percutaneous biopsy underline the importance of these precautions [8,10].

The majority of tumors can be diagnosed with the aid of fine needle aspiration cytology; however, there is a great deal of cytomorphologic overlap with other tumors. It is important to remember the distinctive cytologic characteristics of pheochromocytoma, which are also uncommon findings. Prominent nucleoli in cells can mimic or suggest adenocarcinoma, as well as resemble malignant glandular cells. Pheochromocytomas may exhibit dense, granular, eosinophilic cytoplasm, indicating squamous differentiation. Nuclear pleomorphism and significant nuclear atypia are common features of neuroendocrine tumors. Indeed, this characteristic has been employed as a criterion to differentiate paraganglioma from adenocarcinoma, since the latter is less likely to exhibit this kind of cellular pleomorphism within the same tumor. For an accurate cytologic diagnosis on an FNA specimen, the precise anatomic location of the tumor is most important [7].

The presence of hypertension with symptoms of adrenergic surge should alert the clinician to the tumors of the paragangliomas family [9]. The clinical manifestations of pheochromocytoma are protean but are generally dominated by signs and symptoms of catecholamine hypersecretion. Therefore, for the diagnosis of these lesions, urinary estimation of catecholamines and their metabolites is a more reliable biochemical investigation than plasma estimation. Computed tomography (CT) scan is the radiological investigation of choice for localization. I131- or I123-labeled meta-iodobenzylguanidine scan is indicated in extra-adrenal tumors to rule out metastases [10]. FNA and biopsy of pheochromocytoma are usually contraindicated in literature, but when done with the combination of image guidance, keeping in mind its complications, they can establish the diagnosis [11].

The majority of the literature of cytological features of pheochromocytoma describes a hemorrhagic background mostly, as aspiration produces bloody but cellular smears with numerous poorly cohesive cells. These are moderately sized tumor cells that are polygonal, pleomorphic, and occasionally spindle cells. Characteristically, there is a delicate, moderate to abundant amount of granular eosinophilic cytoplasm in which red granules can be seen in May-Grunwald Giemsa (MGG) stain and round to oval nuclei with mild to moderate anisokaryosis. Intranuclear cytoplasmic inclusions are usually easily found, similar to this case. The chromatin is described as fine and dusty, with a salt and pepper pattern, and nucleoli may or may not be prominent. Histologically, tumor cells are composed of intermediate to large polygonal cells arranged in alveolar, trabecular, and solid patterns. In tumors with alveolar arrangements, the group of tumor cells is surrounded by a capillary-rich framework and sustentacular cells, giving a characteristic Zellballen pattern [12,13].

According to Bhargava et al. [14], diagnosis of extra-adrenal paragangliomas on FNAC is mostly incidental and not an intentional event. Considering these facts, FNAC has a limited role in diagnosing or suggesting a neuroendocrine tumor in an undiagnosed and unsuspected retroperitoneal mass and should not be recommended in routine practice. Apart from the risk of hemorrhage, FNAC can also lead to hypertensive crises, which can be fatal if not managed properly and immediately [8].

However, because adrenal FNA can cause complications such as bleeding and a hypertensive crisis, it is best to rule out pheochromocytoma in people who have never had cancer [15]. Pheochromocytoma was not suspected in our patient since the adrenal nodule was clinically considered to be a renal carcinoma metastasis. As a result, catecholamine and its metabolite levels were not evaluated. Our patient's FNA procedure went without a hitch, which supports FNA of pheochromocytoma not necessarily being contraindicated in literature, but aspiration must be performed in a well-equipped area necessary to control a pheochromocytoma crisis [16].

## Conclusions

While FNA is not universally contraindicated in pheochromocytoma, its use carries significant risk and must be approached with extreme caution. Given the potential for catecholamine surge leading to hypertensive crisis and other life-threatening complications, FNA should be considered only in exceptional situations where noninvasive investigations fail to establish the diagnosis and the result would directly influence management. Any such procedure must be performed in a well-equipped setting with continuous hemodynamic monitoring and immediate availability of emergency antihypertensive support. Importantly, a strong clinical or radiologic suspicion of pheochromocytoma should always prompt prior biochemical confirmation of catecholamine excess, reinforcing that biochemical evaluation remains the cornerstone of diagnosis and that invasive procedures should be avoided unless absolutely necessary.
